# Multimodal analysis of disinformation and misinformation

**DOI:** 10.1098/rsos.230964

**Published:** 2023-12-20

**Authors:** Anna Wilson, Seb Wilkes, Yayoi Teramoto, Scott Hale

**Affiliations:** ^1^ Oxford School of Global and Area Studies, University of Oxford, Oxford OX1 2JD, UK; ^2^ Department of Physics, University of Oxford, Oxford, UK; ^3^ Landmark Information Group, Reading, UK; ^4^ Oxford Internet Institute, University of Oxford, Oxford, UK

**Keywords:** literature review, multimodal dis-/misinformation, qualitative analysis, qualitative analysis, machine learning

## Abstract

The use of disinformation and misinformation campaigns in the media has attracted much attention from academics and policy-makers. Multimodal analysis or the analysis of two or more semiotic systems—language, gestures, images, sounds, among others—in their interrelation and interaction is essential to understanding dis-/misinformation efforts because most human communication goes beyond just words. There is a confluence of many disciplines (e.g. computer science, linguistics, political science, communication studies) that are developing methods and analytical models of multimodal communication. This literature review brings research strands from these disciplines together, providing a map of the multi- and interdisciplinary landscape for multimodal analysis of dis-/misinformation. It records the substantial growth starting from the second quarter of 2020—the start of the COVID-19 epidemic in Western Europe—in the number of studies on multimodal dis-/misinformation coming from the field of computer science. The review examines that category of studies in more detail. Finally, the review identifies gaps in multimodal research on dis-/misinformation and suggests ways to bridge these gaps including future cross-disciplinary research directions. Our review provides scholars from different disciplines working on dis-/misinformation with a much needed bird's-eye view of the rapidly emerging research of multimodal dis-/misinformation.

## 1. Introduction

As disinformation and misinformation proliferate in everyday society, so does the collective need to fully understand the nature of this threat so that effective counter-dis-/misinformation strategies can be developed.

Studies using frameworks from psychology, cognitive science, political science, computer science, and beyond, demonstrate that dis-/misinformation presents a threat to individuals and societies when it negatively impacts their behaviours and decision-making. Misinformation has existed long before the Internet [1–3], but the Internet has lowered the costs of producing and re-sharing misinformation [4], contributed to political polarization, and created environments where people often do not stop to reflect about the accuracy of what they see online [2,3]. Dis-/misinformation can impact people’s beliefs and attitudes [5] including how they vote in political contests [6]. Concretely, dis-/misinformation can contribute to vaccine avoidance [7,8] and worsen health outcomes [9]. Dis-/misinformation can also hinder policy action to counter-act climate change [10], suppress collective cooperation [11] and impact the defence and security of democratic societies [12–14].

Disinformation and misinformation is spread via textual, audio and visual modalities, or multimodally through various combinations of these modalities. While studies of textual dis-/misinformation are more common, there is rapidly growing appreciation of the necessity to do more research on the visual content of dis-/misinformation (see, e.g. the critical review on visual misinformation and the attempt at the systematization of the latter in [15]). As summarized by Heley *et al.* [15], research shows visual information is, among other things, more persuasive and manipulative and more effective in prompting emotional responses. Visual information is remembered for longer and often affects people negatively in more covert ways, as visual manipulations tend to be easily overlooked. In an attempt to answer the question of what visual misinformation is, Heley *et al.* [15] demonstrate that visual content can either be misinformation in itself or constitute part of what we define as multimodal misinformation, e.g. when visual content is contextualized in a certain manner by the accompanying text or speech to become misinformation. Khan *et al.* [16] similarly use ‘multimodal’ to label this category of dis-/misinformation drawing upon semiotics. Much of the visual content in dis-/misinformation offered via traditional and new media comes in the form of video which more often than not constitutes multimodal dis-/misinformation. Dis-/misinformation in the form of videos is viewed as more convincing, believable, and is shared more then text and audio versions. For example, Sundar *et al.* [17] empirically demonstrate that video misinformation is perceived as more credible than text or audio: especially for issues where a person is less informed or interested, ‘seeing is believing’. Video, of course, relies on both audio and visual channels, and the role of audio itself should not be underestimated. For example, humans cannot reliably detect speech deepfakes [18]. The above-mentioned body of research demonstrates the need to examine dis-/misinformation from a multimodal perspective. Our review engages with studies focusing on more than one modality—textual, audio, visual—and treats all of them as multimodal dis-/misinformation regardless of whether the authors refer to them as fakes, manipulation, propaganda, disinformation, misinformation, malinformation, conspiracy theories or other similar terms.

Disinformation and misinformation are successful when they are more subtle, more memorable, more entertaining and more believable than factual information. Multimodal strategies are often employed as weapons of communication to conceal persuasive and manipulative intent, and increase interest, thereby making false information more appealing to audiences, more memorable and hence more effective.

Our three main research questions are informed by the UK’s *Online Harms White Paper of April 2019*, the EU’s *Action Plan against Disinformation of December 2018*, the joint statement *Managing the COVID-19 Infodemic of September 2020* and our prior policy engagement. They aim to help our understanding of what the field of multimodal research on dis-/misinformation is: What are the dynamics of its development? What is its disciplinary and interdisciplinary nature? What are the theories and methods that drive the development of the field? Are the data used reliable? What modalities are investigated? In what ways does multimodal analysis add value according to the authors whose studies we examine?

We draw the map of research trends prevailing in the field and identify niches which need to be filled. We offer potential future directions for multimodal research on dis-/misinformation in an attempt to analyse and tackle dis-/misinformation as one of the greatest challenges of the twenty-first century.

We set out the problem description and the details of data collection and our methodology in §2. We present the analysis of data in §3. After 2020, the number of papers in scope grew substantially—mostly as a result of papers from computer science. Firstly, this required narrowing our focus to papers explicitly considering multimodal analysis in §4. Secondly, we also examined computer science papers in more detail in the same section. The rapid growth of computer science studies of multimodal dis-/misinformation after 2020 motivated us to divide our study and its presentation in this paper into two stages: before 2020, and after the first quarter of 2020. Stage 1 of our study is presented in §3, and Stage 2 is presented in §4. Lastly, we discuss and conclude our review in §5.

### 1.1. What is disinformation and misinformation?

We define disinformation in line with the UK’s *Online Harms White Paper of April 2019* [19], as the deliberate dissemination of information that is false, with the express aim to mislead or obfuscate. Misinformation is similar, but lacks intentionality. Note that disinformation can lead others into misinformation.

It is worth noting that we avoid the use of the term ‘Fake News’, given its politicized nature. However, a large number of authors refer to ‘Fake News’ as the topic of their research, which we see as a subcategory of dis-/misinformation.

### 1.2. What is multimodal communication? Why is it important to analyse it?

Most human communication is more than just words: it is multimodal. Humans use visual, verbal and sound modes in order to communicate. What do intonation, facial expression, gesture and body language add to a communicated message? How do people use emojis, images and videos to communicate on social media? How are television or cinema audiences directed, or manipulated, by the producers’ choice of timing, settings, camera movement, etc.? How do political entities frame the same event from different angles by foregrounding certain aspects of multimodal communication? Ultimately, how can an understanding of the underlying mechanisms of multimodal communication inform how we live our lives? If we are to understand human communication in its full complexity then we need to answer questions such as these.

Multimodal communication plays an important role in many areas of research from linguistics to political science, from business to computer science. On the one hand there is a need to develop analytical models and methods for multimodal communication, combined with large multimodal datasets on which these models and methods can be tested. Naturally, this requires an ecosystem suitable for the collection of such datasets, along with pipelines for semi-automatic and automatic annotation. On the other hand, there is a further need to build capacity in the research methods suitable for multimodal communication, and then to deploy this in evidence-based policy settings and other knowledge exchange activities.

It is worth noting that some authors may have different definitions for modality. Though these definitions generally refer to distinct qualities (e.g. treatments in medicine), this paper requires that a mode be relevant to human communication.

### 1.3. What is multimodal analysis of dis-/misinformation? Why is it important? What are the challenges?

Multimodal analysis is the analysis of two or more modalities—language, gestures, images, sounds, among others—in their interrelation and interaction. The study of one modality in isolation overlooks the complexity of communication practices in terms of how textual, aural, linguistic, spatial and visual resources are integrated to create a single discourse or communication unit. It is especially evident in the case of media. Multimodal analysis reveals and interprets the use of several modalities in composing media messages. It assesses how messages are transformed into tools of persuasion and manipulation. The latter is of particular relevance to the study of dis-/misinformation communication. The importance of researching dis-/misinformation in a multimodal fashion and at scale has been established thorough research on dis-/misinformation at the International Multimodal Communication Centre at the University of Oxford.^1^ The ongoing research shows that there is a need to analyse dis-/misinformation not just in the sense of verifiably incorrect information (via fact-checking), but also in the form of certain types of framing of information which aim to mislead or obfuscate less explicitly but more insidiously [20]. Such framings are more often than not achieved through multimodal communication. Multimodal analysis reveals the detailed composition of multimodal media messages—certain combinations of visual, audio and textual information—and their relation to socio-political, cultural and historical contexts. It reveals what makes these messages manipulative. The IMCC research has engaged, among other topics, with multimodal dis-/misinformation communicated by the Russian state and targeting international and domestic audiences (see, e.g. Uhrig *et al.* [21] on the scaling up of multimodal analysis of RT shows in English).

Consider just one example: the analysis of a Russian TV talk-show ‘Pravo Golosa’ (2012–2019). The show is broadcast in Russian and is representative of Russian disinformation communicated in covert ways. It discusses domestic and international news and invites guests with alternative (anti-Kremlin) viewpoints for the sole purpose of discrediting them in subtle, clever ways. On the surface, the programme seems to be supporting an exchange of views, arguments and constructive critique. In reality, the anchor (and the whole production crew) uses a wide range of multimodal strategies to ensure that the alternative viewpoints are discredited, but the manner of doing so is almost invisible to the untrained eye. At the same time, disinformation is communicated in an engaging and memorable way.

In one episode from February 2016 discussing Ukrainian politics and the outcomes of Maidan (the Ukrainian ‘revolution’ of 2014), the anchor relies on frames and conceptual metaphors such as Self–Other, Russia is a Great Power, Ukraine is Sick, and Anarchy and Banditry in Ukraine versus Law and Order in Russia. The construction of discrediting (disinformation) viewpoints is rooted in cultural and historical knowledge shared by Russians and Ukrainians. The anchor employs a range of manipulation techniques grounded in the co-presence of speech and co-speech gestures. For example, he uses deictic gestures to construct a strong overarching message: Ukrainian People Are Self versus Ukrainian Politicians Are Other. He first works hard to present the (purposefully selected) pro-Ukrainian panelists as incompetent, untrustworthy and corrupt. He then encourages the audience to associate their impression of the panel with all Ukrainian politicians and the whole of Ukrainian politics. Every time the anchor talks about Ukrainian authorities and politicians in a subtly discrediting way, he gestures towards the Ukrainian panel (the hand gesture pointing to the ‘other’ ). By contrast, the anchor brings his hand(s) close to his chest (the ‘self’ gesture normally accompanying words like: I, self, myself, my own, etc.) when talking about the Ukrainian people (on body-directed gestures see [22,23]).

The show also uses co-speech metaphoric gestures as tools of manipulation. These include examples of hand gestures adding crucially important dimensions to the meaning. For example, the purely verbal and relatively neutral ‘You changed the [Ukrainian] regime’ is transformed via a metaphorical hand gesture made by the anchor into the stronger ‘You overthrew the regime’ with the implication that ‘overthrowing’ was illegal/illegitimate. The anchor also speaks with a specific intonation, which adds the epistemic stance of certainty, and makes the statement incorporating the gestural ‘overthrow’ more categorical. Furthermore, the anchor uses gestures and corporal cues to construct a viewpoint of a strong and powerful Russia versus a weak Ukraine. One example is when the anchor adjusts his posture and moves as though he is preparing for a real physical fist fight. The accompanying hand gestures can be labelled as ‘bring it on’.

These conceptualizations—Ukrainian people as SELF versus Ukrainian government/politicians as OTHER, Ukrainian Political Regime is Illegitimate, and Weak Ukraine versus Strong Russia in its ‘bring it on’ aspect—have continued to be communicated by Russian state media as disinformation until the present time. Those conceptualisations were among the key ones on which Putin relied in his speeches of 21 and 24 February 2022 when justifying the start of Russia’s ’special military operation’ (the war against Ukraine) and more recently in e.g. Putin’s speech at the ceremony of annexation of Ukrainian regions on 30 September 2022.

Experimental psychologists interpret the use of such co-speech gestures as lowering the cognitive load on the audience and distributing semantic information across language and visual inputs. They also emphasize that once the information conveyed by both language and co-speech gesture has been processed by the viewer, the influence of it cannot be undone. For example, Kelly *et al.* [24] showed that gestures cannot be ignored, even when people are asked to just make judgements on speech. Gesture–speech integration is ‘automatic.’ The viewer does not register what parts of the information are conveyed by which mode, and would not think of the work done by a particular gesture as a manipulative technique. The main implication for disinformation communication behind the examples here is that on the linguistic level the information sounds reasonably neutral, yet when combined with co-speech gesture, it is enriched with semantic nuances that make the overall resulting message into successful disinformation. Such manipulation techniques allow Russia (or other hostile states) to make its disinformation covert—more subtle yet powerful—and to avoid accountability for the disinformation it communicates, and make it very difficult for viewers to spot that they are being manipulated or understand how they are being manipulated [23]. The text-only approaches which currently prevail in dis-/misinformation analysis are missing the information communicated multimodally, which makes the results of text-only analyses of dis-/misinformation much less reliable.

The very nature of multimodal analysis necessitates the development and application of multidisciplinary and interdisciplinary approaches—a task which is far from trivial.

Starting from the 1990s scholars in such disciplines and research areas as communication and advertising [25–27], semiotics, education studies, linguistics and discourse analysis [28–30] have been proposing various accounts of multimodal research. These accounts prepare the ground for the development of today’s scholarship on multimodal communication analysis. The topics that scholars focus on include: multimodal research accounts of metaphor and other figurative devices [31]; critical analysis of multimodal discourse [32]; multimodal analysis of large datasets [33]; multimodal argumentation and rhetoric in media [34]; multimodal viewpoint analysis [35,36], language and gesture researched from the perspectives of cognitive linguistics [37–40], psychology of language [41–43] and semiotics [44,45].

Computer scientists have recently become more interested in multimodal analysis too. Correctly dealing with multimodal inputs is of huge importance to the field, particularly machine learning (ML) research. Applications range from sophisticated robotics to disinformation detection. Multimodal analysis in computer science has been buoyed by recent advancements in both hardware and ML techniques.

Although there are studies successfully researching multimodal communication, there are also many missed opportunities stemming from the lack of interdisciplinary approaches. There is also a lack of studies focusing on analysing ecologically valid multimodal data in context and at scale. One meaningful initiative which had attempted to bridge the latter gap is the Red Hen Lab [46].^2^ Bearing in mind the broader situation with multimodal communication research, we engage with research on multimodal dis-/misinformation more specifically.

### 1.4. Research questions

The importance of considering disinformation through a multimodal lens, and its highly interdisciplinary nature, motivate our research questions:

**RQ1**: To what extent have studies across different disciplines engaged with multimodal analysis of dis-/misinformation? What is the extent of interdisciplinary practices within the field?
**RQ2**: What methods and data are used by the studies of multimodal dis-/misinformation? What modalities do studies engage with? What value does multimodal analysis add according to the studies? Is multimodal analysis of dis-/misinformation a well-formed research area? What are the challenges this field is facing?
**RQ3**: What types of multimodal studies of dis-/information add value to the field and in what ways?

In the process of investigating the above, we will present the map of research trends observed within the field. The ultimate goal is not to dictate specifics, but instead draw the research landscape for multimodal analysis of dis-/misinformation, suggest a future research agenda for the field and inspire best working practices and approaches.

Our review analysis is divided into two stages: publications before the second quarter of 2020 (Stage 1), and publications from the second quarter of 2020 to August 2022 (Stage 2). Stage 2 reflects the rapid increase in the interest in multimodal analysis by computer scientists.

## 2. Material and methods

### 2.1. Keyword selection and database searches

**Figure 1.**
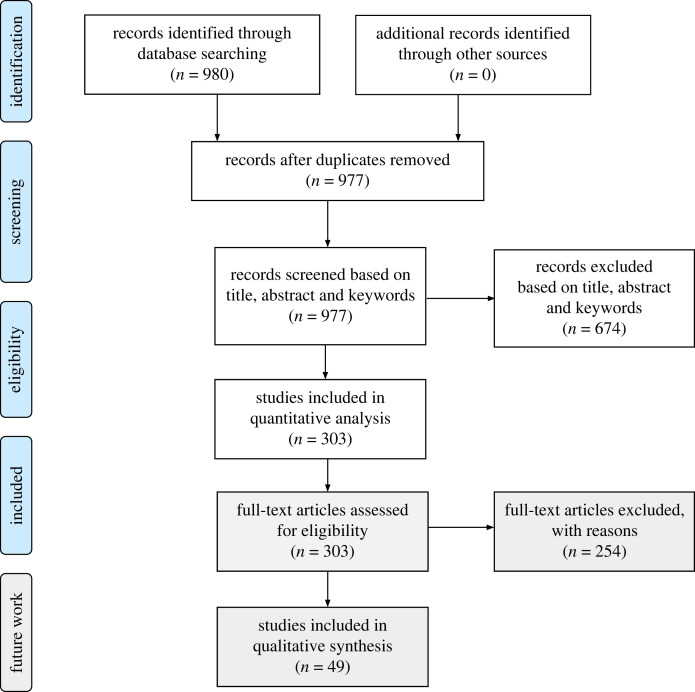
PRISMA statement. The number of records included at each step are shown.

The reporting strategy follows the PRISMA (Preferred Reporting Items for Systematic Reviews and Meta-Analyses; http://www.prisma-statement.org) reporting the checklist approach to systematic literature reviews (figure 1) [47].

The current review was interested in papers with three characteristics. First, they had to study disinformation or misinformation. Second, they had to focus on more than one modality (e.g. image and text or sound and image or video and text). Finally, the articles had to focus on traditional broadcast or social media.

Scopus, a database of peer-reviewed articles in a variety of fields including science, technology, medicine, social sciences and arts and humanities, was used to search for records published using the following search criteria:

LANGUAGE (English)

AND

TITLE-ABS-KEY (‘Fake news’ OR disinformation OR misinformation OR ‘stance on stance’ OR ‘meta-stance’ OR propaganda OR ‘viewpoint construction’ OR ‘viewpoint analysis’ OR ‘multimodal viewpoint’)

AND

(image* OR gesture* OR facial OR video* OR multimodal OR multi-modal OR multimedia OR (sound AND mode) OR (visual AND mode) OR gaze OR ‘eye tracking’ OR prosody OR ‘discourse analysis’ OR ‘media discourse’ OR ‘political discourse’ OR non-verbal OR verbal OR vocal OR non-linguistic OR ‘camera movement’ OR laughter OR applause OR (stress AND (lexical OR word OR sentential OR phonetic)) OR kinetic OR corporal)

AND

(social OR media OR twitter OR facebook OR youtube OR wechat OR weibo OR livejournal OR orkut OR ‘VK’ OR VKontakte OR telegram OR WhatsApp OR Instagram OR reddit OR Wikipedia OR ‘news article’ OR online OR TV OR broadcast)

This yielded 980 results on Scopus in April 2020.

### 2.2. Screening

The results were manually screened based on the title, abstract and keywords. Articles were excluded if they were not written in English, were a collection of conference proceedings rather than individual articles, or were review articles. Furthermore, abstracts were excluded if they focused on a single modality or if they were not on data taken from social media, advertising or broadcast media (e.g. television, radio, newspaper). Likewise, articles were only included if they were about events in the period 1900 to the present. Articles studying strategies to reduce misinformation or disinformation like education programmes and public health campaigns were excluded, as were studies on the misinformation effect and memory malleability in the context of witness reliability.

Eligible articles were then divided into two categories. The first category contained abstracts that explicitly mentioned the use of multimodal analysis to study the content of dis-/misinformation on social media or broadcast media. The second category included abstracts that looked at mis/disinformation more broadly (e.g. responses to misinformation) or articles that looked at political propaganda with no explicit evaluation of the veracity of the information contained in the materials studied.

### 2.3. Identifying the field of research

In order to identify what disciplines were represented in our dataset, we used the ‘All Science Journal Classification’ (ASJC) database published by Scopus, which assigns one or more subject and sub-subject code to journals in their database. Eighty-nine of the sources in our dataset—primarily conference proceedings and books—had to be labelled manually as they were not in the Scopus database. For visualization purposes, we grouped the sub-subjects into intermediate categories.

### 2.4. Topic modelling

Topic modelling using latent Dirichlet allocation (LDA) was performed on the abstracts. This technique uses word frequencies to identify topics and was used to gain a better understanding of the themes that were being studied. We performed this topic modelling on the full list of eligible articles as defined in the ‘Screening’ section. The number of topics was chosen using coherence scores.

### 2.5. Qualitative analysis

A total of 101 full-text articles was analysed qualitatively. We assessed these based on five criteria, namely: (i) whether they were written in English (irrespective of whether they had an English-language abstract); (ii) whether the full text was available online; (iii) whether grounded in original research; (iv) whether they focused on more than one modality; (v) whether they focused on misinformation, disinformation, ‘fake news’ or propaganda. Forty-nine of the 101 articles met these criteria and as a result were included in the final in-depth qualitative analysis.

The full text of all papers was reviewed qualitatively and information about each was added into an extraction table covering the following points:

(i) Bibliographical information
(ii) Data used (source of the data, how they were obtained, how dis-/misinformation was identified)
(iii) Modalities studied
(iv) Methodology used
(v) Working definition of dis-/misinformation
(vi) Main findings
(vii) Value of multimodal analysis according to the authors
(viii) Ethical or social challenges raised by authors

### 2.6. Co-citation network analysis

We created a co-citation network of the 49 articles included in the full text qualitative analysis. The reference list for each article was obtained from its SCOPUS entry, and the journal names were extracted using regular expressions. Each journal was a node in the network, and edges were drawn between journals that cited each other. Network analysis was carried out using networkx and community. Community detection was carried out using the Louvain algorithm with a minimum community size of 10. In addition to looking at citations between journals, we also obtained what subjects were citing each other, using the SCOPUS database of journals. Of the 2043 journals and publication venues, only 1616 were in the SCOPUS database as books and conference proceedings were not in the database.

## 3. Stage 1: Results

The eligible records were identified using the title, abstract and keywords. In total, 303 articles focused on more than one modality, focused on dis-/misinformation and used data collected from social media or broadcast media. A subset of these records (*n* = 101) focused specifically on analysing the content of dis-/misinformation. Most of the analysis presented here focuses on the entire set of eligible records, but some comparisons are drawn between the eligible set and the content-specific subset.

**Figure 2.**
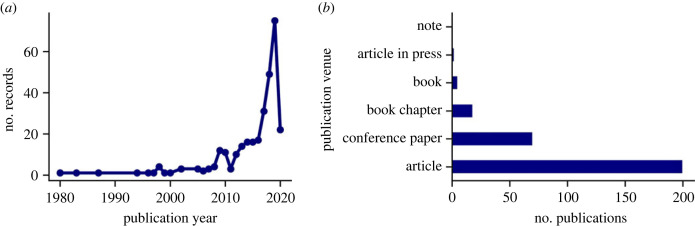
(*a*) Number of records published each year (1900 to 31 March 2020) within scope of Stage 1. (*b*) The number of records for each type of document.

As expected, the number of studies focusing on dis-/misinformation has increased in the last decade (figure 2*a*). This increase appears to happen in two distinct phases. The first increase from 2008 to 2016 is probably due to the increasing popularity of online media including social media platforms like Twitter, Facebook and Instagram as well as online platforms like YouTube. The second phase is much steeper and started from 2016 onward, and is probably due to the increasing sensationalization of ‘fake news’ and online misinformation. The apparent dip in 2020 is due to the fact that the data collected for Stage 1 only includes the first quarter of that year.

**Figure 3.**
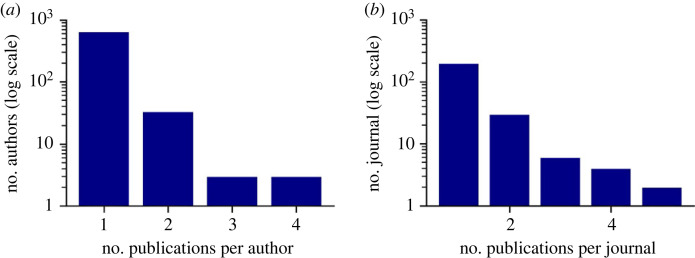
The number of records by the same author (*a*) or records published in the same source (*b*).

Most of the eligible records were published as research articles in journals as well as part of conference proceedings (figure 2*b*). There was a total of 703 unique authors, but most of these authors were only in one publication. Only three authors appeared in three records and three authors in four records (figure 3*a*). Likewise, there were 241 unique publication venues, but very few of them appeared more than once (figure 3*b*). Both of these results suggest that multimodal analysis on dis-/misinformation in media is not concentrated in a select number of established research communities, but rather publications are spread out across many journals and conferences, and few researchers have (yet) done multiple studies on this topic.

**Figure 4.**
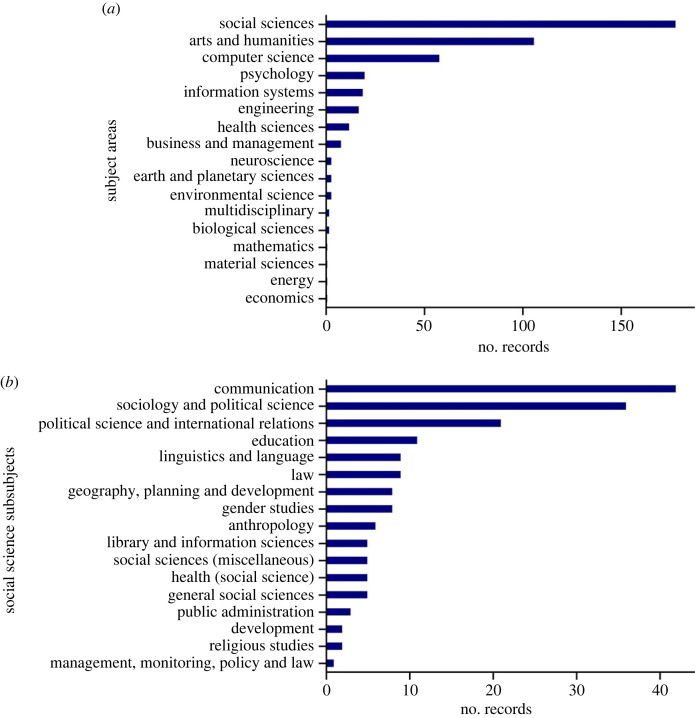
Subject breakdown. The breakdown of subjects for all eligible records (*a*) and the breakdown into sub-subjects for the records in the social sciences (*b*).

The absence of journals with a large number of records or authors that published a lot in the area suggested that multimodal analysis of dis-/misinformation in media does not have a single research community. This suggested that the eligible sources could belong to a diverse set of disciplines. The 241 sources were cross-referenced with Scopus’ source database that has the primary subjects and sub-subjects published in journals. For conference proceedings that were not in the database, the subjects and sub-subjects were hand-coded. Most of the records were published in the social sciences as well as the arts and humanities, but there were a lot of other fields represented including computer science and life sciences (figure 4*a*). Furthermore, for the records in the social sciences, there was also a diverse set of sub-subjects with the primary areas being communications and political science (figure 4*b*). While multimodality is a ‘hot topic’ in computer science (especially within natural language processing and computer vision), our analysis found that within Stage 1 few computer science publications on multimodality were specifically about dis-/misinformation.

**Figure 5.**
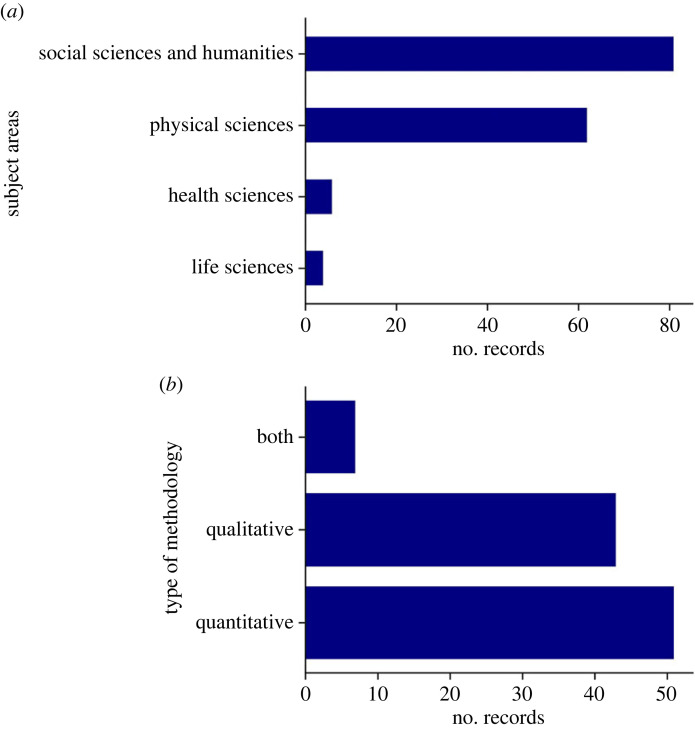
Methodology. The distribution of subjects for content-specific eligible records (*a*), and the type of methodology used in these records (*b*).

Finally, in addition to understanding the disciplinary contributions, the methodology used for the multimodal analysis was studied. This analysis focused only on the subset of records with a focus on analysing dis-/misinformation multimodal content. The strategy for multimodal analysis was determined to be qualitative (e.g. discourse analysis) or quantitative (e.g. deep learning) or both. The breakdown of subject areas for this subset was similar to the breakdown of subject areas of the full set of eligible records (figure 5*a*). Even though social sciences and humanities were the primary subject areas represented, more than half of the articles used quantitative methods (figure 5*b*). This suggests that several of the studies in the social sciences used quantitative methods in addition to the records published in engineering science or computer science journals. Nonetheless, there are still very few papers that attempt to combine both qualitative and quantitative approaches to multimodal analysis.

### 3.1. Topic modelling

**Figure 6.**
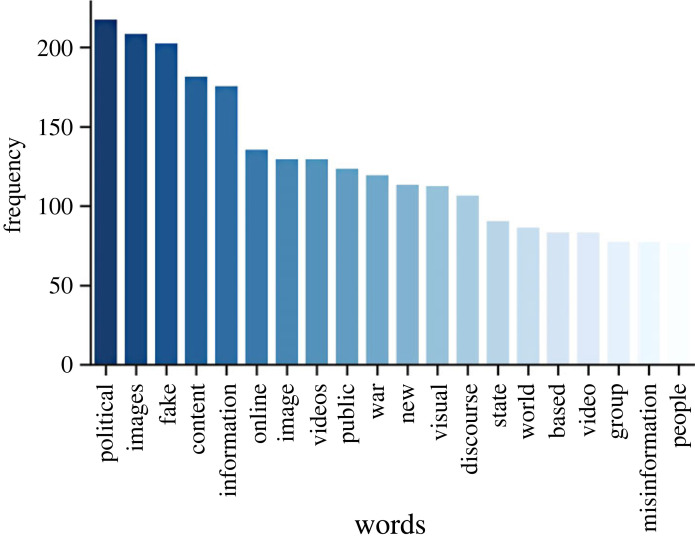
The 20 most common words in the abstracts after stop words were removed.

Topic modelling was then applied to understand what topics studies on dis-/misinformation in online or broadcast media were focused on. The most common words in the dataset after removal of the most common words in English included many of the search terms used in Scopus like ‘news’ and ‘propaganda’ (figure 6). However, there were words like ‘political’, ‘war’ and ‘state’ that suggest that a lot of the research focuses on dis-/misinformation in the context of political events or conflicts.

**Figure 7.**
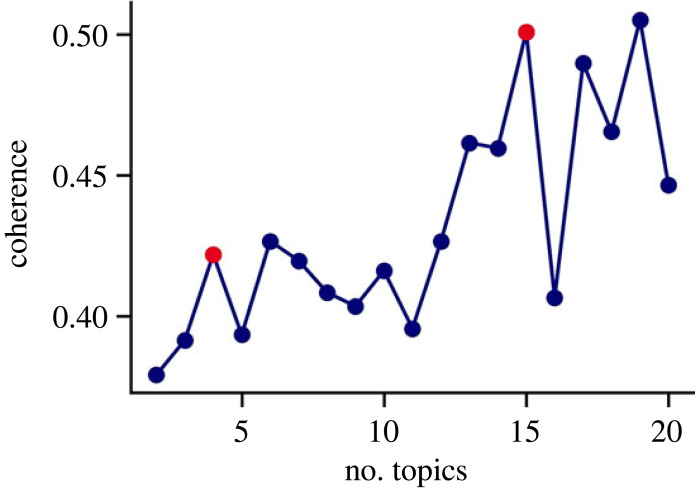
Coherence scores for LDA models for different numbers of topics (minimum 2). The red-highlighted points indicate possible choices of topic numbers.

Our study trained a LDA topic model on the abstracts of our subjects. LDA is an established method in topic modelling that uses the frequency of words in each abstract to iteratively assign words to topics and topics to abstracts. In order to determine the number of topics to use, the coherence score was used, which is a measure of the quality of the topics that were found. Typically, the coherence score plateaus or falls after reaching a good number of topics. Based on the scores (figure 7), the rest of the analysis was carried out for *N* = 4 topics.

Table 1 lists the topics detected by the LDA algorithm for *N* = 4 topics, with the top words assigned to each topic and a qualitative interpretation of what each topic may correspond to. Three of the topics correspond to types of misinformation, where Topic 1 corresponded specifically to online misinformation and Topics 0 and 3 corresponded to propaganda for political and religious purposes. Topic 2 appeared to have a mix of different themes including gender, war and religion.

**Table 1.** Topic modelling results for *N* = 4 topics.

topic no.	top words	annotation
0	propaganda ISIS media war political social also online radio group	religious propaganda
1	news fake social media content information videos images online misinformation	online/social media content
2	women war public American Muslim popular year one first anti-communist	gender; religion; war
3	media political propaganda social discourse images visual communication new news	political propaganda

**Figure 8.**
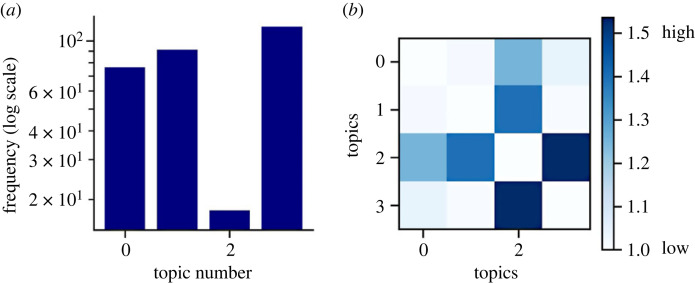
The frequency with which each topic was the primary topic in an abstract (*a*) and the normalized frequency with which topics co-occurred with other topics (*b*).

Each abstract will contain words that belong to each topic, so each abstract can be assigned one or more topics that mostly represent it. The predominant topic for each abstract was obtained, and the three topics related to misinformation were similarly represented in the abstracts (figure 8*a*). Topic 2 was the main topic for only 18 abstracts, but appeared as a topic in 39 abstracts, which may suggest that this topic contains words that refer to multiple aspects of the dis-/misinformation content being studied. This is confirmed by looking at the frequency with which topics co-occur with each other normalized to their relative frequency. Topic 2 is the only one that seems to co-occur with the other topics (figure 8*b*). Among the three dis-/misinformation topics, the two propaganda-related topics (Topics 0 and 3) had a slightly higher co-occurrence compared to the overlap between Topic 1 and either Topic 0 or Topic 3.

**Figure 9.**
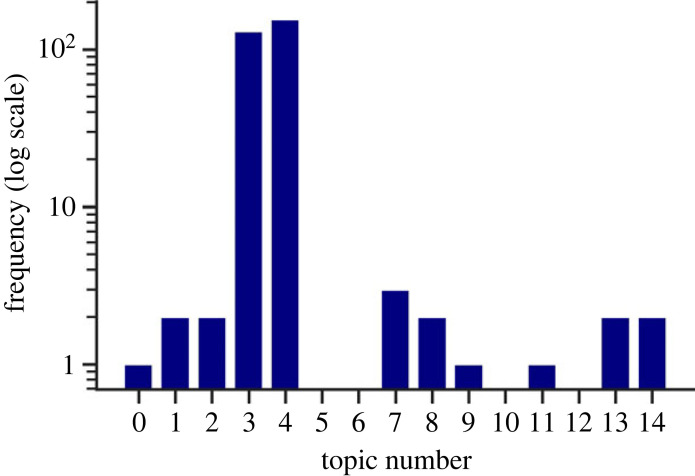
The frequency with which each topic was the primary topic in an abstract.

We looked at the distribution of different disciplines within each topic (figure 9). There is a clear disciplinary divide in the vocabulary employed to refer to dis-/misinformation, because while articles published in social sciences and humanities journals spoke about it in terms of words related to politics and propaganda, articles published in the physical sciences, like computer science, tended to focus on social media and use terms like ‘fake news’.

### 3.2. Qualitative analysis

Next, a more in-depth qualitative analysis was carried out on the full text of 49 articles. We engaged with the parameters for the analysis outlined in §2.6 above, which are translated here in the subheadings in this section of our paper. Our engagement produced the following results.

#### 3.2.1. Methodology employed by articles

*Methods*. Most studies used either quantitative (*n* = 20) or qualitative (*n* = 22) methods, but around 10% of them used a mixture of both (*n* = 7).

*Data*. Apart from the types of methods used, there was a marked difference in the data sources employed by quantitative papers as opposed to qualitative or mixed methods papers. Over half of the quantitative papers (*n* = 12) used publicly available datasets in their analysis, whereas the qualitative and mixed method papers all collected data specific to their proposed research questions. Two datasets that were used by multiple papers include the Weibo dataset [48] and the MediaEval Twitter dataset [49]. Both of these are freely available and individual items have been labelled as rumour/non-rumour or fake/true, respectively.

*Combinations of modalities*. We found most articles focused on two modalities rather than three (noting that articles analysing only one mode are not within the scope of our review). Forty articles focused on the analysis of two modalities. Of these, 38 focused on image and text [20,48,50–85]. Two articles focused on sound and image [86,87].

Nine articles focused on the analysis of three modalities. Seven focused on text, sound/music and video (including dance performance): [88–94]. Two articles focused on text, image, video (incl. sound): [95,96].

#### 3.2.2. Qualitative papers: theoretical frameworks and methods used

Our meta-analysis revealed quantitative methods were used by 20 articles while qualitative methods were used by 22 articles; seven papers additionally used a combination of quantitative and qualitative analyses. We used qualitative analysis to establish which theories and methods were employed by 22 studies that researched dis-/misinformation through qualitative methods. Our meta-analysis revealed:

(I) Eleven papers used a form of discourse analysis as their core method as rooted in linguistics, rhetoric and/or social semiotics, while combining their discourse analysis with a number of other theories and methods key to their analysis of content. Of those:
  — two papers used multimodal discourse analysis (MDA) as based on work done by G.R. Kress and T. van Leeuwen ([50,65]; with literature cited);
  — three papers used multimodal critical discourse analysis (MCDA) as based on works by D. Machin, A. Mayr, R. Wodak, T. van Leeuwen and G.R. Kress ([62,71,89]; with literature cited);
  — one paper used critical discourse analysis (CDA) drawing upon frameworks offered by T.A. van Dijk and by N. Fairclough in combination with a genre analysis approach by J.M. Swales ([81]; with literature cited);
  — one paper used systemic functional multimodal discourse analysis (SF-MDA) based on work by O’Halloran 2008 to analyse iconisation ([54]; with literature cited)
  — one paper used ‘diatextual’ analysis—a rhetorical approach to discourse analysis developed by the authors ([54]; with literature cited);
  — one paper used a rhetorical approach to discourse analysis based on M. Bakhtin’s notion of carnival ([90]; with literature cited);
  — one paper used relied on the work done by T.A. van Dijk and P. Bourdieu for their discourse analysis ([73]; with literature cited);
  — one paper drew upon S. Hall’s work for the analysis of media power and class power and on N. Cook’s work for analysis of music ([94]; with literature cited).
(II) Seven papers engaged in content analysis while using approaches originating from political communication, sociology, history, philosophy and anthropology, and anchored in:
  — philosophy of language (epistemic activism, resistance, friction) based on work by J. Stanley’s work ([74]; with literature cited);
  — framing analysis (grounded theory) based on works by R. Entman, D. Scheufele, D. Tewksbury & S.D. Reese ([97]; with literature cited);
  — human geography as rooted in anthropology, history and sociology ([85]; with literature cited);
  — cultural sociology (grounded theory) ([68]; with literature cited);
  — theories of cognitive dissonance, parasocial interaction, social identification (grounded theory) ([93]; with literature cited);
  — analysis by considering theories of gendered framing [67];
  — an ethnographic technique, which is document-driven and across multiple sites, is used by Krafft & Donovan [53], based on the principles of Geiger & Ribes 2011.
(III) Six papers did not have a clearly defined theoretical and methodological framework [66,77,83,86,87,92].

Out of 22 papers only 12 engaged in multimodal analysis of content which relied on a defined theoretical and methodological framework. Papers from (I) were grounded in frameworks which allow for fine-grained and evidence-based analyses of social interaction and associated power relations, whereas papers from (II) relied rather on intuition of analysts for their interpretation of language and visual inputs in message communication while focusing more on the analysis of context than rather than content. Papers from (III) presented descriptive analyses not rooted in any clearly defined frameworks for qualitative analyses. Out of 22 papers only four from (I) relied on an approach rooted in linguistics and social semiotics for multimodal analysis of discourse and communication of dis/misinformation. We note that despite large bodies of research done on multimodal communication in cognitive and corpus linguistics as well as experimental psychology, both disciplinary areas have been conspicuously absent from multimodal research on dis-/misinformation.

#### 3.2.3. Quantitative papers: theoretical frameworks and methods used

By contrast to the previous section, for quantitative papers (*n* = 20) we observed less variety in the theoretical and methodological approaches that they used. We found that the main approaches of each quantitative paper fell into the following five categories, within which there was much more uniformity compared to the diversity of qualitative papers in each of three categories above:

(I) Fifteen papers used some form of black-box statistical analysis; a cursory analysis yields:
  — twelve papers chose to train multimodal networks with supervised learning;
  — two papers [60,63] employed unsupervised learning techniques for their training;
  — one paper [80] directly leveraged existing AI tools to extract pertinent details from a dataset.
(II) Interpretable statistical methods were applied to six papers in order to analyse the authors’ data
  — conducting and analysing surveys were the focus of three papers; of these, two papers [52,56] went beyond simple statistics by using a hierarchical/multilevel regression analysis scheme;
  — one paper [91] developed a vector method of categorizing social media posts based on their reliability and consistency;
  — results from a psychology experiment [20] were analysed using various statistical methods.
(III) The creation of a multimodal disinformation database was the focus of two papers [55,72].
(IV) One paper [84] included network and spatial distribution analyses to investigate how disinformation spread.
(V) One paper [95] discussed the development of software to perform fact-checking.

We note that except for three papers in (II), all the publications above originated from the field of computer science. The majority of quantitative papers tried to automate the detection of false news by the training of ML algorithms. On the other hand, only one paper attempted to study the actual properties of disinformation using ML. This disparity is indicative of a wider issue. It indicates that advanced quantitative methods to detect multimodal disinformation are being prioritized over investigating multimodal features of disinformation; there is no historical corpus to suggest the latter is a solved problem. At the same time, the narrow spread of research highlights the possibility of missed opportunities to answer broader research questions, particularly those outside computer science. An interdisciplinary approach may avoid this.

Though these quantitative papers evidently engaged with the topic of disinformation, rarely did they investigate the roles that multiple modalities play. This means despite the clear interest from computer science, it is predominantly other disciplines that drive forward the theoretical understanding of multimodal disinformation.

#### 3.2.4. Instances where papers employed both qualitative and quantitative methods

Incorporating both quantitative and qualitative methods probably indicates the development of pre-existing manual approaches. Seven papers were found to do both; these can be considered to fall within three broad categories:

(I) Expanding qualitative analyses with quantitative approaches
  — one paper [65] used the approach of multimodal discourse analysis by C. Jewitt to annotate a subset of the dataset studied; then a software-based multimodal analyser was used to expand this analysis across their dataset;
  — one paper used systemic functional multimodal discourse analysis (SF-MDA) based on work by O’Halloran followed a similar approach as above by using the same software [78];
  — one paper first has health experts manually annotate videos [88], and after extracting for a range of video features (such as a transcript or acoustic features) a ML model is constructed to detect health misinformation within videos.
(II) Attempting to clarify quantitative results by subsequently applying qualitative analysis
  — after automatic coding of URLs was used to determine the types of media shared on WhatsApp, one paper [96] manually reviewed a randomized subset of the collected data to provide a more fine-grained understanding of the URL content;
  — one paper that created a multiclass classification network examined a collection of the model’s outputs; from this they could determine what categories of material their model was able to better recognize [69].
(III) A collection of disparate methods used as part of a single topic of investigation
  — one paper manually sought to descriptively categorize the types of shared content, before engaging in a network analysis of the spread of such content [51];
  — one psychology paper employed a range of statistical analyses on their experimental results which was followed by qualitative insights gained from subject interviews [61].

While six papers above had computer scientist authors, three of these papers had interdisciplinary authorship. In the context of all the computer science papers we analysed, interdisciplinary authorship accounted for only around 15% of the work. Two of these papers included arts and humanities authors, while the third featured contributions from the medical sciences. This interdisciplinarity unlocked the possibility of new work.

#### 3.2.5. Definitions of dis-/misinformation

All of the articles analysed looked at an aspect of how information can be manipulated or distorted in online or broadcast media in the form of misinformation, disinformation, or more broadly propaganda or bias. More than half of the articles (*n* = 33) assumed the definition of misinformation implicitly and did not provide a definition. Only five articles provided a definition of disinformation and four articles provided a definition of misinformation, while eight articles provided a definition of fake news.

Of the articles that provided a definition of misinformation and disinformation, only one article [64] used the same definition as used in this paper. While all of the variations of the definition of disinformation alluded to the intentionality of the agents spreading false information, the definitions of misinformation were more variable. Unlike the definition adopted by this report, none of them noted the unintentional nature of misinformation. This suggests little progress in the field of understanding the mechanisms and subtleties of (multimodal) misinformation.

For papers that consider detection of dis-/misinformation, it is crucial to define clearly and fully the problem being investigated. The provision of clear and full definitions of dis-/misinformation is an issue the research field needs to address going forward.

#### 3.2.6. Value of multimodal analysis according to the authors of studies

Six out of 49 articles did not discuss the value of multimodal analysis. The articles which addressed this question did so with a varying degree of explicitness. Those which were more explicit noted that multimodal analyses add value in that, for example:

— a multimodal approach which considers the functions of language and images and/or videos together has the potential to shed further light on understanding the construction and impact of propaganda [78], with the visual modality typically being key for communicating across countries and cultures [82];
— visual input changes the way a person is perceived [20];
— ignoring visual content on social media loses a lot of the content [80];
— the text, sound, dance, visuals and context interact as a unit to convey multiple layers of meaning [94];
— multimodal considerations of multimodal data boost detection rates when compared to unimodal approaches [48].

#### 3.2.7. The overall findings of the published studies

The articles differed in terms of how clearly they presented their findings and achievements and also in terms of the domains within which those findings and achievements fell, namely: (i) the development of theoretical and methodological approaches for multimodal analysis of dis-/misinformation including but not limited to the detection of fakes; (ii) the creation of new multimodal datasets; (iii) insights from qualitative and/or quantitative analyses of multimodal data (e.g. discourses and media contents) of a certain kind. The broad variety of theories, methodologies, and type of data used by the studies considered prevented us from going deeper into the analysis of their findings and achievements here, but the majority of authors emphasized the importance of analysing visual and sound data in addition to textual data to improve the accuracy and validity of dis-/misinformation and propaganda research including that on the detection of fakes. At the same time the authors commented on the challenges which such multimodal research presents. We engage in a deeper analysis of the findings of the subcategory of the studies reviewed below in Section ‘Stage 2: Zooming into multimodal disinformation and misinformation publications after March 2020’—those mainly originated from the computer science.

#### 3.2.8. Ethical or social challenges raised by the authors of studies

Out of 49 articles, only one explicitly engaged with of an ethical challenge [73]. The paper, having observed and followed ‘far-right and neo-facist’ social media posts, touched upon the ethical issues of researching violent and extremist content. It also discussed how researchers can be protected and whether to reveal the identities of people calling for violence in the context of the wider issue of the invasion of privacy. For the purposes of their study, the author chose to anonymize all the published results.

Research on multimodal dis-/misinformation ought to engage better, and more explicitly, with ethical and social challenges. Such engagement ultimately translates into informing government policies, among bringing other social benefits and needs to form part of the research field.

### 3.3. Co-citation networks

The network community detection is good at recovering groupings that we had identified based on the methods and research questions of the papers. The co-citation analysis and visualization was performed on journals that were cited a minimum of 5 times.

Then we looked at what fields were citing each other (results shown in table 2). The subjects are taken from Scopus. With the exception of journals in life sciences (which in our dataset were journals in cognitive neuroscience), journals in one discipline predominantly cited articles published in journals in the same discipline. For example, 80% of the citations within social science and humanities journals are to other journals in the social sciences and humanities. Overall, social science journals made up at least 25% of all citations regardless of the discipline of the citing journal.

**Table 2.** The tendency for a subject to cite another subject is shown between four subject categories. Specifically, the proportion, for each subject, of citations for the *citing* journal to the *cited* journal is shown in the right-most column.

citing journal subject	cited journal subject	normalized % citations
social sciences and humanities (*n* = 351)	social sciences and humanities	80
social sciences and humanities	physical sciences	11
social sciences and humanities	life sciences	0
social sciences and humanities	health sciences	9
physical sciences (*n* = 33)	social sciences and humanities	33
physical sciences	physical sciences	61
physical sciences	life sciences	6
physical sciences	health sciences	0
life sciences (*n* = 39)	social sciences and humanities	38
life sciences	physical sciences	31
life sciences	life sciences	0
life sciences	health sciences	31
health sciences (*n* = 4)	social sciences and humanities	25
health sciences	physical sciences	0
health sciences	life sciences	0
health sciences	health sciences	75

## 4. Stage 2: zooming into multimodal disinformation and misinformation publications after March 2020

The growth of papers falling within our search parameters has an exponential profile, as shown in figure 2. Hence, individually sifting through each paper would become unfeasible. Moreover, as COVID-19 has shaped many research interests, we felt it was appropriate to set March 2020 as a threshold to more narrowly consider only publications that explicitly aimed to research multimodal dis-/misinformation. To do this, the steps of §2 were repeated in August 2022, except that we additionally filtered out any papers that did not contain the (case-insensitive) key words {MULTIMODAL,MULTI−MODAL} in either the title or abstract. This reduced the list to *n*_0_ = 133. Similar to Stage 1, papers that were not directly related to multimodal dis-/misinformation were manually excluded: this gave a final *n*_f_ = 78. We report on our observations relevant to advancing the field, highlighting best practices.

### 4.1. The rise of computer science since second quarter of 2020

**Figure 10.**
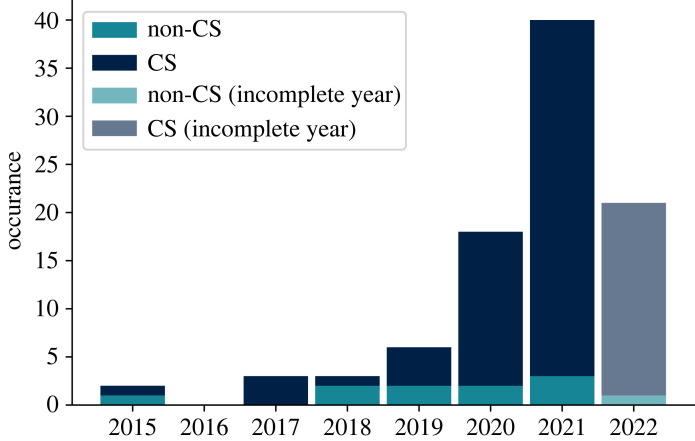
This chart compares the number of CS versus non CS publications across both Stages 1 and 2 using the stricter selection criteria outlined in Stage 2. All papers were screened to check that they were applicable to dis-/misinformation; the earliest example dated from 2015.

From 2020 onward, computer science (CS) clearly became the significant majority of all these publications considered (figure 10); in fact it largely accounts for the overall growth in publications. Evidently this particular shift to CS merits further analysis. Out of the *n*_f_ = 78, *n*_cs_ = 73 had CS authors, from a range of international institutions (table 3). This section aims to motivate the direction of future work.

**Table 3.** The ‘intensity’ of country’s research into multimodal disinformation was estimated by summing the instances of each participating institution across all papers, and normalizing by the number of citeable documents for that host country [98].

country	research intensity (×10^4^)
Qatar	3.961
Bulgaria	2.992
Vietnam	1.155
India	0.927
Spain	0.576
Singapore	0.433
China	0.426
USA	0.306
UK	0.257

#### 4.1.1. Automatic detection of multimodal dis-/misinformation with machine learning

Advances in ML algorithms, coupled with decreased costs to access computing hardware, has led to many more computer scientists applying ML to new tasks. As such, the stated purpose of an overwhelming 90.4% of papers within this Stage 2 review set proposed a new method for the detection of multimodal dis-/misinformation. Applying ML to multimodal dis-/misinformation has garnered such interest that within our review set two new workshops in this area had emerged. A number of papers were from the workshop ‘De-Factify’—concerning multimodal fact-checking and hate-speech detection—and another paper was submitted as part of the workshop ‘MAD2022’ which focused on multimedia disinformation.

**Figure 11.**
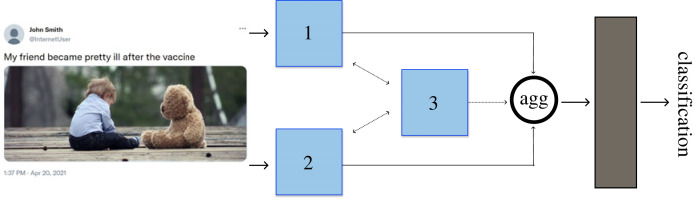
This is a general schematic of a bimodal disinformation detector. The blue boxes labelled 1 and 2 are modules that process each modality of the input data. The role of module 3 is to pass information, no matter how complex, between modalities. The outputs of these modules are first aggregated before then being passed into a black box classifier; concatenation and ‘multi-layer perceptrons’ were the most commonly observed ML methods, respectively.

Usefully combining multiple distinct inputs for use in neural networks is challenging. This is because the various inputs can be weak (uninformative), or may have a strong interdependence. As multimodal disinformation varies widely, so does the salience of each modality, or their interactions. Consequently, Chen *et al.* [99] and Song *et al.* [100] show that including multiple modalities, without additional handling, can increase detection noise. This suggests that dynamically altering the importance of each modality is crucial. Figure 11 depicts a generalized multimodal disinformation detector; a dynamic implementation is able to vary the weighting between modules 1–3 depending on the input data. If the weightings are frozen after training, this naïvely assumes that there is minimal distributional difference between the training data and the real world, which is often unwarranted [101]. A total of 8 papers did this. Unfortunately, a further 23 papers (31.5%) in our Stage 2 review set had either a weighting of 0 for module 3’s connections, or performed ‘late fusion’ of single-modality classifier outputs. Neither of these methods is sufficient to infer the general properties of disinformation, which can depend strongly on the multimodal interactions.

Finding more suitable misclassification penalties (i.e. ‘loss functions’) can itself prove effective. Three papers [102–104] attempted to address the heterogeneity of the disinformation landscape by enforcing orthogonality between distinct news events. This was done by employing an ‘adversarial approach’ to the loss function during training. The competition between the generator and classifier networks helped to capture differences between domain-specific and domain-independent features. This approach requires defining, or categorizing, these domains *a priori*.

#### 4.1.2. Attempts to analyse the models and their decisions

Most papers engaged with our question of unimodality versus multimodality by disabling a modality input—a process referred to as ‘ablation’. However, these tended to show only small improvements from the inclusion of images. This may be in part be explained by the strengths of natural language processing algorithms. Some authors additionally performed qualitative analysis of their ablation, but often the examples found, where a multimodal paradigm prevailed, had minimal linguistic rationale. We point to Wu *et al.* [105] as a clear example where the authors’ model had captured modality-specific cues.

Understanding why models respond in the way they do is one of the goals of developing ‘explainable AI’ or ‘XAI’. Given the potential complexity of multimodal disinformation, if such detectors are to be seriously considered in the real world, incorporating XAI methods may become a key requirement. The three main approaches we encountered were:

(I) Direct analysis of the detection model; only one paper [106] explicitly did this, which provided insight into what aspects of the data their model was reacting to
(II) Examination of the dataset(s) properties
  — mainly constrained to dataset creation papers (see next section), though estimates for biases are not always performed;
  — the models may be configured to present data statistics, for instance presenting the levels of inter-modality interaction [99], the discordance of each modality [107], or the model’s modality weightings [108].
(III) Visualizing the decision; visualizing data and the model can offer insight to researchers, but aside from [109] this was rarely attempted

Particularly for (II) and (III), domain experts can be invaluable for finding clues and patterns; this again suggests an interdisciplinary approach may provide new insights. Lastly, we note that for (II), there has been no work systematically exploring biases (and if these biases may be themselves be multimodal) and their effects on disinformation detection.

#### 4.1.3. ‘Fake News’: definitions, prevalence and classification consequences

As seen in the papers in Stage 1, key words relating to disinformation are often not defined, only accounting for 37% of this subset. This may be partly explained by widespread adoption of the term ‘Fake News’. This is synonymous with binary classification—the dominant paradigm. By contrast, only seven papers (9.6%) acted to classify in at least three ways [102,110–116]; definitions were introduced to justify their classification objectives. A general disinformation detector cannot fall within the scope of a binary classifier. It is still possible for a binary classifier to able to accurately categorize a subset of disinformation; defining this type of disinformation is then a practical necessity.

### 4.2. Multimodal data, its properties and how it was used

**Figure 12.**
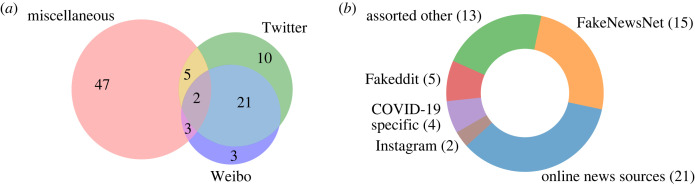
Shown here is where the data originates and how these sources mix. The breakdowns were chosen to highlight the sorts of disinformation studied. A source was counted if the paper’s data source was distinct (e.g. datasets ‘X’ and ‘Y’ count as two). We counted 91 instances of sources, hence an average mixing of 1.25 sources per paper—largely independent. (*a*) Depicting the mixing of data sources and (*b*) breakdown of ‘Miscellaneous’ in (*a*).

There were five non-CS papers. Of these, full video featured heavily; two papers applied multimodal analysis [113,117] to investigate the video content and messaging, whereas one psychology paper investigated the effects of flagging deepfakes on subjects [118]. On the other hand, only a couple of CS papers went beyond two modalities: one focused on examining sequences of images and their captions from YouTube [119] and one paper on TikTok went further still by also incorporating audio [120]. Some papers stated metadata as an additional modality, but as justified in §1.2 this would not be counted in this paper. The sources of data used by papers were not extensive; the levels of data-source mixing are depicted in figure 12*a*.

We saw a number of detection papers expressing regret at the lack of accessible datasets. The stated aims of seven papers were to add to the range of available training data. The main challenge is labelling large quantities of data. For datasets focusing on collating online news articles, two papers chose to label by quantifying the publishers ‘credibility’ based on results from fact-checking organizations [121,122]. This is problematic though as disinformation is complex and can be intermingled within legitimate news and claims; see, for example [123]. Using automatic claim matching, two papers sought to create multilingual datasets [116,124]. One paper manually verified samples of the data studied, which exclusively focused on Reddit posts [111]. Another approach is to augment [125] or synthesize data [126]. Only one paper considered the threat of the dataset studied, filtering out results that were too straightforward by using an adversarial approach [126]. Moreover, the authors tested their dataset on real humans as a benchmark—an important step, only otherwise fulfilled in [112]—finding both that humans struggled to distinguish between fake and real examples and that their detector performed at a comparable level. It should be noted that while many papers conducted analyses of the datasets’ textual content, only two papers [110,127] additionally collected statistics on their images.

Only two papers attempted to test their detection models on live unseen data. For instance, Wang *et al.* [128] scraped and manually labelled COVID-related Instagram posts once a day for a month, and obtained very similar classification results to their initial offline run.

The early detection of disinformation is vital in limiting its harm, but unseen disinformation can prove challenging for detection (zero-shot classification). If instead a few initial examples within a nascent category are allowed to be manually labelled, this can allow for ‘few-shot classification’. To this end, one paper presented a meta-learning approach [129], which aimed to jointly learn category and global features as they arose. Similarly, one paper held back training examples based on their post time to mimic real life conditions [130], testing their models performance for different delay times. Overall, we found limited engagement with temporal features of disinformation. In particular, no papers considered the longer term evolution of disinformation, nor presented analysis on any distribution shifts.

### 4.3. Interdisciplinary and non-computer science publications

The stricter corpus requirements left few papers outside CS. Though the sample size is small, the remaining non-CS papers neither notably deviated in approach nor in quality from those papers studied in the Stage 1.

In one paper [127] theories of communication both informed the quantitative approach and yielded insight into the statistical features of their dataset. Two CS papers [110,112] stood out for their use of interdisciplinary methods. These papers both studied memes—inherently multimodal objects—and drew from methods outside CS for data annotation and analysis methods, as well as informing their use of quantitative methods.

## 5. Discussion and conclusion

Our meta-study has shed some light on how research on multimodal dis-/misinformation in media communication has been evolving in the past 20 years. Our division of meta analysis into two stages—before and after the second quarter of 2020—has reflected changes in the development of the research area due to the start of the COVID pandemic in the second quarter of 2020, namely the prevalence of multimodal research of dis-/misinformation originating from computer science from 2020 onwards.

Our meta-analysis in Stage 1 identified 303 articles researching dis-/misinformation in media while also focusing on more than one modality. 101 out of those 303 performed multimodal analysis of the content of dis-/misinformation. Our further in-depth qualitative analysis focused on full texts of 49 articles that met our inclusion criteria.

Our meta-analysis has revealed that there is no single disciplinary or cross-disciplinary community employing multimodal analysis to study dis-/misinformation. Most authors and publication venues appeared only once in our dataset, which suggested that multimodal analysis of dis-/misinformation was not the main subject of study of any given researcher or research community. The diversity of disciplines, from which articles originated—from the social sciences and computer science to management, engineering and health sciences—further pointed to the lack of an established research community with the focus on multimodal dis-/misinformation. Our topic modelling analysis of the abstracts revealed a disciplinary split. Abstracts from articles published in the social sciences and humanities scored highly on the propaganda topics and low on the online media topic, whereas those published in the physical sciences (including computer science) scored highly on the online media topic but not on the propaganda topics. This dissociation suggested that a barrier to establishing a cross-disciplinary research community was a lack of common focus, terminology and definition of dis-/misinformation.

Our in-depth analysis of 49 full-text articles revealed a further binary opposition, this time related to the research focus and method used: articles which employed quantitative methods were primarily interested in creating frameworks to detect whether a particular news item or social media post was ‘fake news’ or not. By contrast, articles using qualitative methods mainly focused on propaganda analysis, as well as multimodal strategies of persuasion and manipulation in media discourse and communication. Only 7 articles out of 49 articles used a mix of qualitative and quantitative methods. Those originated from disciplines which would traditionally use a qualitative approach to analysis. Quantitative methods were used by them to scale up and further support qualitative analysis.

The majority of studies engaged with the question of the added value of multimodal analysis as well as of the challenges of developing and applying theories and methods suitable for multimodal analysis of dis-misinformation. For many papers these engagements constituted part of the studies’ findings.

Our analysis demonstrated that only one paper out of 49 engaged explicitly with the question of ethical and social challenges of multimodal research on dis-/misinformation. We argue that these challenges need to be addressed by the field better in the future.

Furthermore, more than half of those 49 articles did not contain definitions of misinformation, disinformation, fake news or propaganda. If present, the definitions varied across studies. This suggested the lack of uniform understanding of the objects of study to which those terms refer.

Our analysis in Stage 1 revealed that research on multimodal dis-/misinformation would benefit from the development of one established area, with clear definitions of research objects, a goal to address ethical and social challenges, a unified terminology and cross-disciplinary methodological practices. Cross-disciplinary practices would benefit not only disciplines that traditionally use qualitative methods, but also those which would traditionally rely on quantitative methods.

Although we observed a general increase in studies focusing on multimodal dis-/misinformation in 2008 and 2016, it is only from the second quarter of 2020 that we observed a rapid increase in computer science studies on multimodal dis-/misinformation.

As our meta-analysis of Stage 2 demonstrated that there was no notable change in the research on multimodal dis-/misinformation which originated from disciplines other than computer science. It is computer science studies which became the driver for the explosive growth of research on multimodal dis-/misinformation. That growth came with scholars across many cultures engaging in multimodal dis-/misinformation research.

Those changes motivated the emerging need to understand the extent to which the choices in ML techniques, more specifically, were informed by knowledge originating from humanities and social sciences. The in-depth examination of full-text articles at Stage 2 revealed positive dynamics in how the computer science studies under consideration addressed the complexities associated with the analysis of multiple modalities. However, while in aggregate these papers employed a range of multimodal strategies, no single paper brought these together. The missed opportunities were driven to a considerable extent by the lack of interdisciplinary approach to analysis. We also identified clear research gaps, such as the limited work on the temporal nature of multimodal dis-/misinformation.

Our meta-study at Stages 1 and 2 demonstrated that most studies, regardless of discipline, focused on two modalities rather than three. This may be explained by scholars’ intention to keep analysis more straightforward, but also by the use of pre-prepared data. Especially within computer science, most studies used existing datasets rather than constructing their own.

For articles analysed in both Stages 1 and 2, we observed no engagement with questions about how dis-/misinformation evolves over time, including shifts in distribution patterns. We consider that this is at least partly due to a lack of studies which employ true interdisciplinary approaches to investigation of dis-/misinformation.

Nonetheless, single-discipline papers still brought value to the overall study of multimodal dis-/misinformation. In addition to moving beyond text-only approaches, papers that provided definitions of dis-/misinformation, those constructing novel datasets—especially video—and those which used a combination of qualitative and quantitative methods were particularly valuable.

Our meta-analysis has revealed the potential for computer science techniques to aid theories of multimodal dis-/misinformation communication originating from the range of disciplines in humanities and social sciences, and scale up qualitative analysis to provide statistical validity. Explainable AI could be a large help in this regard, especially if developed with social science and humanities expertise. More interaction across the humanities and social sciences with computer science could enable further development of AI methods for multimodal analysis of dis-/misinformation. This would require more interdisciplinary research and collaboration to ensure better understanding of the findings originating from the disciplines of humanities and social sciences.

Our meta-analysis has also demonstrated the challenges of conducting multimodal analysis of dis-/misinformation and the nature of the associated gap in research. The gap manifests itself through the absence of a coherent body of multimodal research on disinformation and misinformation. The divide between different disciplines and research interests in the field was present throughout our analysis including the topic modelling of abstracts, the co-citation analysis and the manual qualitative analysis on the full text of 127 articles (49 full-text articles were analysed at Stage 1 and 78 articles at Stage 2).

With the advent of accessible computing technology, large scale quantitative analyses constitute a clear new avenue for research into multimodal disinformation and misinformation. Indeed, we observed a recent uptake of this approach; however, efforts to leverage these methods have largely been confined to computer science. This has resulted in many missed research opportunities and even has manifested in experimental design and analysis that is not motivated by theories of multimodal communication. Moving forward, creating a more unified research landscape is needed, which will require the development of unified terminology and definitions suitable for analysis of multimodal dis-/misinformation, as well as a conscious effort from scholars to cross boundaries of disciplines. Among other things, interdisciplinarity should enable more studies to focus on video data and as a result to examine three modalities—verbal (text), sound, visual—as opposed to just two modalities. Further development of interdisciplinary approaches to the analysis of multimodal dis-/misinformation should also empower researchers to investigate at scale subtle manipulation which forms a large part of dis-/misinformation communication, but is more difficult to research than ’fakes’.

## Data Availability

This article has no additional data.

## Footnotes

1 https://imcc.web.ox.ac.uk/projects.

2 Homepage at https://www.redhenlab.org.

## References

[1] Altay S, Berriche M, Acerbi A. 2023 Misinformation on misinformation: conceptual and methodological challenges. Soc. Media Soc. **9**, 1-13.

[2] Pennycook G, Rand DG. 2021 The psychology of fake news. Trends Cogn. Sci. **25**, 388-402. (10.1016/j.tics.2021.02.007) 33736957

[3] Pantazi M, Hale S, Klein O. 2021 Social and cognitive aspects of the vulnerability to political misinformation. Pol. Psychol. **42**, 267-304. (10.1111/pops.12797)

[4] Verrall N, Mason D. 2018 The taming of the shrewd. RUSI J. **163**, 20-28. (10.1080/03071847.2018.1445169)

[5] Lewandowsky S, Ecker UK, Cook J. 2017 Beyond misinformation: understanding and coping with the ‘Post-Truth’ Era. J. Appl. Res. Mem. Cogn. **6**, 353-369. (10.1016/j.jarmac.2017.07.008)

[6] Vaccari C, Morini M. 2014 The power of smears in two american presidential campaigns. J. Political Mark. **13**, 19-45. (10.1080/15377857.2014.866021)

[7] Jamieson KH, Romer D, Jamieson PE, Winneg KM, Pasek J. 2021 The role of non-COVID-specific and COVID-specific factors in predicting a shift in willingness to vaccinate: a panel study. Proc. Natl Acad. Sci. USA **118**, e2112266118. (10.1073/pnas.2112266118) 34930844 PMC8719857

[8] Pierri F, Perry BL, DeVerna MR, Yang KC, Flammini A, Menczer F, Bryden J. 2022 Online misinformation is linked to early COVID-19 vaccination hesitancy and refusal. Sci. Rep. **12**, 5966. (10.1038/s41598-022-10070-w) PMC9043199 35474313

[9] Ferreira Caceres MM et al. 2022 The impact of misinformation on the COVID-19 pandemic. AIMS Public Health **9**, 262-277. (10.3934/publichealth.2022018) PMC9114791 35634019

[10] Benegal SD, Scruggs LA. 2018 Correcting misinformation about climate change: the impact of partisanship in an experimental setting. Clim. Change **148**, 61-80. (10.1007/s10584-018-2192-4)

[11] Meng Y, Broom M, Li A. 2023 Impact of misinformation in the evolution of collective cooperation on networks. J. R. Soc. Interface **20**, 206. (10.1098/rsif.2023.0295) PMC10522409 37751874

[12] Verrall N, Dunkley M, Gane T, Byrne R. 2019 Dangerous liaisons: a ‘Big Four’ framework that provides a ‘Hint’ to understanding an Adversary’s strategy for influence. RUSI J. **164**, 52-68. (10.1080/03071847.2019.1643255)

[13] Gill R, Goolsby R (eds). 2022 COVID-19 disinformation: a multi-national, whole of society perspective. Cham, Switzerland: Springer Nature.

[14] Verrall N. 2022 COVID-19 disinformation, misinformation and malinformation during the pandemic infodemic: a view from the United Kingdom. In *COVID-19 disinformation: a multi-national, whole of society perspective* (eds R Gill, R Goolsby), pp. 81–112. Cham, Switzerland: Springer Nature.

[15] Heley K, Gaysynsky A, King AJ. 2022 Missing the bigger picture: the need for more research on visual health misinformation. Sci. Commun. **44**, 514-527. (10.1177/10755470221113833) 36082150 PMC9451169

[16] Khan A, Brohman K, Addas S. 2021 The anatomy of ‘fake news’: studying false messages as digital objects. J. Inform. Technol. **37**, 122-143. (10.1177/02683962211037693)

[17] Sundar SS, Molina MD, Cho E. 2021 Seeing is believing: is video modality more powerful in spreading fake news via online messaging apps? J. Comput.-Mediat. Commun. **26**, 301-319. (10.1093/jcmc/zmab010)

[18] Mai KT, Bray S, Davies T, Griffin LD. 2023 Warning: humans cannot reliably detect speech deepfakes. PLoS ONE **18**, 1-20. 10.1371/journal.pone.0285333 PMC10395974 37531336

[19] UK Government. 2019 Online Harms White Paper. See https://www.gov.uk/government/consultations/online-harms-white-paper (Last accessed 21 Nov. 2023).

[20] Ecker UKH, Lewandowsky S, Chang EP, Pillai R. 2014 The effects of subtle misinformation in news headlines. J. Exp. Psychol.: Appl. **20**, 323-335. (10.1037/xap0000028) 25347407

[21] Uhrig P, Payne E, Pavlova I, Burenko I, Dykes N, Baltazan M, Burrows E, Hale S, Torr P, Wilson A. 2023 Studying time conceptualisation via speech, prosody, and hand gesture: interweaving manual and computational methods of analysis. In *Gesture and speech in interaction* (*GeSpIn*) *conference* (eds W Pouw *et al.*). Nijmegen, Netherlands: Max Planck Institute for Psycholinguistics.

[22] Cooperrider K. 2014 Body-directed gestures: pointing to the self and beyond. J. Pragmat. **71**, 1-16. (10.1016/j.pragma.2014.07.003)

[23] Wilson A. 2020 It’s time to do news again. Zeitschrift für Anglistik und Amerikanistik **68**, 379-409. (10.1515/zaa-2020-2016)

[24] Kelly SD, Özyürek A, Maris E. 2010 Two sides of the same coin: speech and gesture mutually interact to enhance comprehension. Psychol. Sci. **21**, 260-267. (10.1177/0956797609357327) 20424055

[25] Kostelnick C, Roberts DD. 1998 Designing visual language: strategies for professional communicators. Boston, MA: Allyn and Bacon.

[26] Messaris P. 1997 Visual persuasion: the role of images in advertising. Thousand Oaks, CA: SAGE Publications.

[27] Scott LM. 1994 Images in advertising: the need for a theory of visual rhetoric. J. Consum. Res. **21**, 252-273. (10.1086/209396)

[28] Nöth W. 1995 Handbook of semiotics. Bloomington, Indiana: Indiana University Press.

[29] Kress GR, van Leeuwen T. 1996 Reading images: the grammar of visual design. New York, NY: Routledge.

[30] Jewitt C. 2009 The Routledge handbook of multimodal analysis. London, UK: Routledge.

[31] Forceville C, Urios-Aparisi E (eds). 2009 Multimodal metaphor. Berlin, Germany: Mouton de Gruyter.

[32] Machin D, Mayr A. 2012 How to do critical discourse analysis: a multimodal introduction. London, UK: SAGE Publications.

[33] Steen F et al. 2018 Toward an infrastructure for data-driven multimodal communication research. Linguist. Vanguard **4**, 20170041. (10.1515/lingvan-2017-0041)

[34] Tseronis A, Forceville C (eds). 2017 Multimodal argumentation and rhetoric in media genres. Philadelphia, PA: John Benjamins Publishing Company.

[35] Dancygier B, Sweetser E (eds). 2012 Viewpoint in language: a multimodal perspective. Cambridge University Press, Cambridge, UK.

[36] Vandelanotte L, Dancygier B. 2017 Multimodal artefacts and the texture of viewpoint. J. Pragmat. **122**, 1-9. (10.1016/j.pragma.2017.10.011)

[37] Cienki A, Müller C (eds). 2008 Metaphor and gesture. Philadelphia, PA: John Benjamins Publishing Company.

[38] Cienki A, Iriskhanova O (eds). 2018 Aspectuality across languages: event construal in speech and gesture. Philadelphia, PA: John Benjamins Publishing Company.

[39] Grishina E. 2017 *Russian gesticulation from a linguistic point of view. Corpus research* [Русская жестикуляция с лингвистической точки зрения: корпусные исследования]. Izdatel’skij dom Jazyk: Jazyki slavjanskoj kul’tury, Moscow.

[40] Turner M, Avelar M, Mendes de Oliveira M. 2019 Blended classic joint attention and multimodal deixis. Signo **44**, 3-9. (10.17058/signo.v44i79.12710)

[41] Hostetter AB. 2011 When do gestures communicate? A meta-analysis. Psychol. Bull. **137**, 297-315. (10.1037/a0022128) 21355631

[42] Beattie G. 2016 Rethinking body language: how hand movements reveal hidden thoughts. New York, NY: Routledge.

[43] Kita S, Alibali MW, Chu M. 2017 How do gestures influence thinking and speaking? The gesture-for-conceptualization hypothesis. Psychol. Rev. **124**, 245. (10.1037/rev0000059) 28240923

[44] Waisman OS. 2010 Body, language and meaning in conflict situations: a semiotic analysis of gesture-word mismatches in Israeli-Jewish and Arab discourse, vol. 62. Philadelphia, PA: John Benjamins Publishing.

[45] Adami E. 2010 ELF and sign-making practices on YouTube: between globalization and specificities. *From international to local English – and back again* (eds R Facchinetti, D Crystal, B Seidlhofer), pp. 235–264. Bern, Switzerland: Peter Lang AG.

[46] Steen FF et al. 2018 Toward an infrastructure for data-driven multimodal communication research. Linguistics Vanguard **4**. (10.1515/lingvan-2017-0041)

[47] Moher D, Liberati A, Tetzlaff J, Altman DG. 2009 Preferred reporting items for systematic reviews and meta-analyses: the PRISMA statement. Ann. Intern. Med. **151**, 264-269. (10.7326/0003-4819-151-4-200908180-00135) 19622511

[48] Jin Z, Cao J, Guo H, Zhang Y, Luo J. 2017 Multimodal fusion with recurrent neural networks for rumor detection on microblogs. In *MM 2017 - Proc. of the 2017 ACM Multimedia Conf.*, Mountain View, CA, 19 October 2017, pp. 795–816. New York, NY: ACM.

[49] Boididou C, Andreadou K, Papadopoulos S, Dang-Nguyen DT, Boato G, Riegler M, Kompatsiaris Y. 2015 Verifying multimedia use at MediaEval 2015. In *CEUR Workshop Proc.: MediaEval Benchmarking Initiative for Multimedia Evaluation*, Wurzen, Germany, 14-15 September 2015, vol. 1436. Aachen, Germany: CEUR-WS.

[50] Kalkina V. 2020 Between humour and public commentary: digital re-appropriation of the soviet propaganda posters as internet memes. J. Creat. Commun. **15**, 131-146. (10.1177/0973258619893780)

[51] Resende G, Melo P, Sousa H, Messias J, Vasconcelos M, Almeida J, Benevenuto F. 2019 (Mis)Information dissemination in WhatsApp: gathering, analyzing and countermeasures. In *The World Wide Web Conference*, San Francisco, CA, 13-19 May 2019, pp. 818–828. New York, NY: ACM.

[52] Hameleers M, Powell TE, Van Der Meer TG, Bos L. 2020 A picture paints a thousand lies? The effects and mechanisms of multimodal disinformation and rebuttals disseminated via social media. Pol. Commun. **37**, 281-301. (10.1080/10584609.2019.1674979)

[53] Krafft PM, Donovan J. 2020 Disinformation by design: the use of evidence collages and platform filtering in a media manipulation campaign. Political Commun. **37**, 194-214. (10.1080/10584609.2019.1686094)

[54] Scardigno R, Mininni G. 2020 The rhetoric side of fake news: a new weapon for anti-politics? World Futures **76**, 81-101. (10.1080/02604027.2019.1703158)

[55] Jindal S, Sood R, Singh R, Vatsa M, Chakraborty T. 2020 NewsBag: a multimodal benchmark dataset for fake news detection. CEUR Workshop Proc. **2560**, 138-145.

[56] Schaewitz L, Kluck JP, Klösters L, Krämer NC. 2020 When is disinformation (in)credible? Experimental findings on message characteristics and individual differences. Mass Commun. Soc. **5436**, 484-509. (10.1080/15205436.2020.1716983)

[57] Vishwakarma DK, Varshney D, Yadav A. 2019 Detection and veracity analysis of fake news via scrapping and authenticating the web search. Cogn. Syst. Res. **58**, 217-229. (10.1016/j.cogsys.2019.07.004)

[58] Qi P, Cao J, Yang T, Guo J, Li J. 2019 Exploiting multi-domain visual information for fake news detection. In *Proc. of IEEE Int. Conf. on Data Mining, ICDM*, Beijing, 8-11 November 2019, pp. 518–527. New York, NY: IEEE. (10.1109/ICDM.2019.00062)

[59] Singhal S, Shah RR, Chakraborty T, Kumaraguru P, Satoh S. 2019 SpotFake: a multi-modal framework for fake news detection. In *2019 IEEE Fifth International Conference on Multimedia Big Data (BigMM)*, Singapore, 11-13 September 2019, pp. 39–47. New York, NY: IEEE. (10.1109/BigMM.2019.00-44)

[60] Cui L, Wang S, Lee D. 2019 SAME: sentiment-aware multi-modal embedding for detecting fake news. In *Proc. of the 2019 IEEE/ACM Int. Conf. on Advances in Social Networks Analysis and Mining*, Vancouver, BC, 27-30 August 2019, pp. 41–46. New York, NY: ACM. (10.1145/3341161.3342894)

[61] Huang D, Zhu Y, Mustafaraj E. 2019 How dependable are ‘first impressions’ to distinguish between real and fake news websites? In *HT 2019 - Proc. of the 30th ACM Conference on Hypertext and Social Media*, Berlin, Germany, 12-16 September 2019, pp. 201–210. New York, NY: ACM. (10.1145/3342220.3343670)

[62] Smith CA. 2019 Weaponized iconoclasm in internet memes featuring the expression ‘Fake News’. Discourse Commun. **13**, 303-319. (10.1177/1750481319835639)

[63] Jaiswal A, Wu Y, Abdalmageed W, Masi I, Natarajan P. 2019 AIRD: adversarial learning framework for image repurposing detection. In *Proc. of the IEEE Computer Society Conf. on Computer Vision and Pattern Recognition*, Long Beach, CA, 15-20 June 2019, pp. 11322–11331. New York, NY: IEEE.

[64] Khattar D, Gupta M, Goud JS, Varma V. 2019 MVAE: multimodal variational autoencoder for fake news detection. In *The Web Conf. 2019 - Proc. of the World Wide Web Conf.e, WWW May 2019*, San Francisco, CA, 13-16 May 2019, pp. 2915–2921. New York, NY: ACM. (10.1145/3308558.3313552)

[65] O’Halloran KL, Tan S, Wignell P, Bateman JA, Pham DS, Grossman M, Moere AV. 2019 Interpreting text and image relations in violent extremist discourse: a mixed methods approach for big data analytics. Terror. Political. Violence **31**, 454-474. (10.1080/09546553.2016.1233871)

[66] Graham AP. 2019 Hostile visual encounters: fighting to control photographic meaning in the DRC’s digital age. Africa **89**, 266-285. (10.1017/S0001972019000056)

[67] Nee RC, De Maio M. 2019 A ‘Presidential Look’? An analysis of gender framing in 2016 persuasive memes of hillary clinton. J. Broadcast. Electron. Media **63**, 304-321. (10.1080/08838151.2019.1620561)

[68] Zhukova E. 2019 Image substitutes and visual fake history: historical images of atrocity of the Ukranian Famine 1932–1933 on social media. Visual Commun. **21**, 3-27. (10.1177/1470357219888673)

[69] Volkova S, Ayton E, Arendt DL, Huang Z, Hutchinson B. 2019 Explaining multimodal deceptive news prediction models. In *Proc. of the 13th Int. AAAI Conf. on Web and Social Media,* Münich, Germany, 11-14 June 2019, pp. 659–662. Washington, DC: AAAI. (10.1609/icwsm.v13i01.3266)

[70] Angiani G, Lombardo G, Balba G, Mordonini M, Fornacciari P, Tomaiuolo M. 2018 Image-based hoax detection. In *Goodtechs '18: Proceedings of the 4th EAI International Conference on Smart Objects and Technologies for Social Good*, Bologna, Italy, 28-30 November 2018, pp 159–164. New York, NY: ACM. (10.1145/3284869.3284903)

[71] Brookes G, Harvey K, Chadborn N, Dening T. 2018 ‘Our Biggest Killer’: multimodal discourse representations of dementia in the british press. Soc. Semiot. **28**, 371-395. (10.1080/10350330.2017.1345111)

[72] Sabir E, Wu Y, Almageed WA, Natarajan P. 2018 Deep Multimodal Image-Repurposing Detection. In *Proceedings of the 26th ACM international conference on Multimedia (MM '18)*. Seoul, 22-26 October 2018, pp. 1337–1345. New York, NY: ACM. (10.1145/3240508.3240707)

[73] DeCook JR. 2018 Memes and symbolic violence: #proudboys and the use of memes for propaganda and the construction of collective identity. Learn. Media Technol. **43**, 485-504. (10.1080/17439884.2018.1544149)

[74] Medina J. 2018 Resisting racist propaganda: distorted visual communication and epistemic activism. South. J. Philos. **56**, 50-75. (10.1111/sjp.12301)

[75] Knshnan S, Chen M. 2018 Identifying tweets with fake news. In *Proc. 2018 IEEE 19th Int. Conf. on Information Reuse and Integration for Data Science, IRI,* vol. 67, Salt Lake City, UT, 6 July 2018, pp. 460–464. New York, NY: IEEE. (10.1109/IRI.2018.00073)

[76] Wang Y, Ma F, Jin Z, Yuan Y, Xun G, Jha K, Su L, Gao J. 2018 EANN: event adversarial neural networks for multi-modal fake news detection. In *Proc. of the 24th ACM SIGKDD Int. Conf. on Knowledge Discovery & Data Mining*, London, UK, 19-23 August 2018, pp. 849–857. New York, NY: ACM. (10.1145/3219819.3219903)

[77] Kasra M, Shen C, O’Brien JF. 2018 Seeing is believing: how people fail to identify fake images on the web. In *Extended Abstracts of the 2018 CHI Conference on Human Factors in Computing Systems*, Paper No.: LBW516. Montreal, Canada, 21-27 April 2018, pp. 1–6. New York, NY: ACM. (10.1145/3170427.3188604)

[78] Tan S, O’Halloran KL, Wignell P, Chai K, Lange R. 2018 A multimodal mixed methods approach for examining recontextualisation patterns of violent extremist images in online media. Discourse Context Media **21**, 18-35. (10.1016/j.dcm.2017.11.004)

[79] Agarwal A, Sehwag A, Singh R, Vatsa M. 2019 Deceiving face presentation attack detection via image transforms. In *Proc. - 2019 IEEE 5th Int. Conf. on Multimedia Big Data, 5 December 2019*, Singapore, 11-13 September 2019, pp. 373–382. New York, NY: IEEE. (10.1109/BigMM.2019.00018)

[80] Dewan P, Suri A, Bharadhwaj V, Mithal A, Kumaraguru P. 2017 Towards understanding crisis events on online social networks through pictures. In *Proc. of the 2017 IEEE/ACM Int. Conf. on Advances in Social Networks Analysis and Mining,* Sydney, Australia 31 July - 3 August 2017, pp. 439–446. New York, NY: IEEE. (10.1145/3110025.3110062)

[81] Amiri M, Hashemi MR, Rezaei J. 2015 The representation of islamophobia: a critical discourse analysis of yahoo news. Int. J. Control Theory Appl. **8**, 599-618. (10.17485/ijst/2015/v8i28/87385)

[82] Seo H. 2014 Visual propaganda in the age of social media: an empirical analysis of twitter images during the 2012 Israeli–Hamas conflict. Vis. Commun. Q. **21**, 150-161. (10.1080/15551393.2014.955501)

[83] Mina AX. 2014 Batman, pandaman and the blind man: a case study in social change memes and internet censorship in China. J. Vis. Cult. **13**, 359-375. (10.1177/1470412914546576)

[84] Gupta A, Lamba H, Kumaraguru P, Joshi A. 2013 Faking sandy: characterizing and identifying fake images on Twitter during Hurricane Sandy. In *WWW 2013 Companion: Proc. of the 22nd Int. Conf. on World Wide Web*, Rio de Janeiro, 13-17 May 2013, pp. 729–736. New York, NY: ACM. (10.1145/2487788.2488033)

[85] Brayshay M, Selwood J. 2002 Dreams, propaganda and harsh realities: landscapes of group settlement in the forest districts of Western Australia in the 1920s. Landsc. Res. **27**, 81-101. (10.1080/01426390220110784)

[86] Deaville J. 2019 Pitched battles: music and sound in anglo-American and german newsreels of world war II. J. Musicol. Res. **38**, 32-43. (10.1080/01411896.2019.1568153)

[87] Khaldarova I, Pantti M. 2016 Fake news: the narrative battle over the Ukranian conflict. Journal. Pract. **10**, 891-901. (10.1080/17512786.2016.1163237)

[88] Hou R, Loeb S, Pérez-Rosas V, Mihalcea R. 2020 Towards automatic detection of misinformation in online medical videos. In *ICMI 2019 - Proc. of the 2019 Int. Conf. on Multimodal Interaction*, Utrecht, The Netherlands, 25-29 October 2020, pp. 235–243. New York, NY: ACM. (10.1145/3340555.3353763)

[89] Mackay RR. 2015 Multimodal legitimation: selling scottish independence. Discourse Soc. **26**, 323-348. (10.1177/0957926514564737)

[90] Meddaugh PM. 2010 Bakhtin, colbert, and the center of discourse: is there no ‘Truthiness’ in Humor? Crit. Stud. Media Commun. **27**, 376-390. (10.1080/15295030903583606)

[91] Kang S, Hwang J, Yu H. 2020 Multi-modal component embedding for fake news detection. In *Proc. of the 2020 14th Int. Conf. on Ubiquitous Information Management and Communication, IMCOM 2020*, Taichung, Taiwan, 3-5 January 2020, pp. 1–6. New York, NY: IEEE. (10.1109/IMCOM48794.2020.9001800)

[92] Meikle G. 2012 ‘Find Out Exactly What to Think–Next!’: Chris Morris, Brass Eye, and journalistic authority. Popular Commun. **10**, 14-26. (10.1080/15405702.2012.638569)

[93] Bradshaw AS, Treise D, Shelton SS, Cretul M, Raisa A, Bajalia A, Peek D. 2020 Propagandizing anti-vaccination: analysis of vaccines revealed documentary series. Vaccine **38**, 2058-2069. (10.1016/j.vaccine.2019.12.027) 31980194

[94] Monte EP. 2017 Romancing the nation, effacing history: reading Kenya through patriotic choral music. Soc. Dyn. **43**, 451-469. (10.1080/02533952.2017.1394648)

[95] Bagade A, Pale A, Sheth S, Agarwal M, Chakrabarti S, Chebrolu K, Sudarshan S. 2020 The Kauwa-Kaate fake news detection system: demo. In *Proceedings of the 7th ACM IKDD CoDS and 25th COMAD (CoDS COMAD 2020),* Hyderabad, India, 5-7 January 2020*,* pp. 302–306. New York, NY: ACM. (10.1145/3371158.3371402)

[96] Machado C, Kira B, Narayanan V, Kollanyi B, Howard PN. 2019 A study of misinformation in WhatsApp groups with a focus on the Brazilian Presidential Elections. In *Companion Proceedings of The 2019 World Wide Web Conference (WWW '19),* San Francisco, CA, 13-17 May 2019, pp. 1013–1019. New York, NY: ACM. (10.1145/3308560.3316738)

[97] Seo H, Ebrahim H. 2016 Visual propaganda on facebook: a comparative analysis of syrian conflicts. Media War Confl. **9**, 227-251. (10.1177/1750635216661648)

[98] Scimago Journal & Country Rank. See https://www.scimagojr.com/countryrank.php?order=itpord=descyear=2020 (Last accessed 18 January 2023).

[99] Chen Y, Li D, Zhang P, Sui J, Lv Q, Tun L, Shang L. 2022 Cross-modal ambiguity learning for multimodal fake news detection. In *Proc. of the ACM Web Conf. 2022*, Lyon, France 25-28 April 2022, pp. 2897–2905. New York, NY: ACM. (10.1145/3485447.3511968)

[100] Song C, Ning N, Zhang Y, Wu B. 2021 A multimodal fake news detection model based on crossmodal attention residual and multichannel convolutional neural networks. Inf. Process. Manag. **58**, 102437. (10.1016/j.ipm.2020.102437)

[101] Torralba A, Efros AA. 2011 Unbiased look at dataset bias. In *CVPR 2011*, Colorado Springs, CO, 22 August 2011, pp. 1521–1528. New York, NY: IEEE. (10.1109/CVPR.2011.5995347)

[102] Song C, Ning N, Zhang Y, Wu B. 2021 Knowledge augmented transformer for adversarial multidomain multiclassification multimodal fake news detection. Neurocomputing **462**, 88-100. (10.1016/j.neucom.2021.07.077)

[103] Wei P, Wu F, Sun Y, Zhou H, Jing XY. 2022 Modality and event adversarial networks for multi-modal fake news detection. IEEE Signal Process Lett. **29**, 1382-1386. (10.1109/LSP.2022.3181893)

[104] Zhang T, Wang D, Chen H, Zeng Z, Guo W, Miao C, Cui L. 2020 BDANN: BERT-based domain adaptation neural network for multi-modal fake news detection. In *2020 Int. Joint Conf. on Neural Networks* (*IJCNN*), Glasgow, UK, 25-27 September 2020, pp. 1–8. New York, NY: IEEE. (10.1109/IJCNN48605.2020.9206973)

[105] Wu Y, Zhan P, Zhang Y, Wang L, Xu Z. 2021 Multimodal fusion with co-attention networks for fake news detection. In *Findings of the Association for Computational Linguistics: ACL-IJCNLP 2021*, Online, 1-6 August 2021, pp. 2560–2569. Stroudsburg, PA: Association for Computational Linguistics.

[106] Ferreira VC, Kundu S, Franca FMG. 2022 Analysis of fake news classification for insight into the roles of different data types. In *2022 IEEE 16th Int. Conf. on Semantic Computing* (*ICSC*), Laguna Hills, CA, 26 January 2022, pp. 75–82. New York, NY: IEEE. (10.1109/ICSC52841.2022.00018)

[107] Singhal S, Dhawan M, Shah RR, Kumaraguru P. 2021 Inter-modality discordance for multimodal fake news detection. In *ACM Multimedia Asia* (MMAsia '21), Gold Coast, Australia, 1-3 December 2021, pp. 1–7. New York, NY: Association for Computing Machinery. (10.1145/3469877.3490614)

[108] Shang L, Kou Z, Zhang Y, Wang D. 2022 A duo-generative approach to explainable multimodal COVID-19 misinformation detection. In *WWW '22: Proc. of the ACM Web Conf. 2022,* Lyon, France, 25-29 April 2022, pp. 3623–3631. New York, NY: Association for Computing Machinery. (10.1145/3485447.3512257)

[109] Wagle V, Kaur K, Kamat P, Patil S, Kotecha K. 2021 Explainable AI for multimodal credibility analysis: case study of online beauty health (Mis)-information. IEEE Access **9**, 127 985-128 022. (10.1109/ACCESS.2021.3111527)

[110] Dimitrov D, Ali BB, Shaar S, Alam F, Silvestri F, Firooz H, Nakov P, Martino GDS. 2021 Detecting propaganda techniques in memes. In *Proc. of the 59th Annual Meeting of the Association for Computational Linguistics and the 11th International Joint Conference on Natural Language Processing* (*Volume 1: Long Papers*), Bangkok, Thailand, 1-6 August 2021, pp. 6603–6617. Stroudsburg, PA: Association for Computational Linguistics. (https://aclanthology.org/2021.acl-long.516)

[111] Nakamura K, Levy S, Wang WY. 2020 Fakeddit: a new multimodal benchmark dataset for fine-grained fake news detection. In *Proc. of The 12th Language Resources and Evaluation Conference*, Marseilles, France, 20-25 March 2020, pp. 6149–6157. Paris, France: European Language Resources Association. (10.48550/arXiv.1911.03854)

[112] Pramanick S, Sharma S, Dimitrov D, Akhtar MS, Nakov P, Chakraborty T. 2021 MOMENTA: a multimodal framework for detecting harmful memes and their targets. In *Findings of the Association for Computational Linguistics: EMNLP 2021*, Punta Cana, Dominican Republic, 7-11 November 2021, pp. 4439–4455. Stroudsburg, PA: Association for Computational Linguistics. (10.18653/v1/2021.findings-emnlp.379)

[113] Gamir-Ŕıos J, Tarullo R, Ibáñez-Cuquerella M. 2021 La desinformació multimodal sobre l’alteritat a Internet. Difusió de boles racistes, xenòfobes i islamòfobes el 2020 [Multimodal disinformation about otherness on the internet. The spread of racist, xenophobic and Islamophobic fake news in 2020]. Anàlisi: Quaderns de Comunicació i Cultura **64**, 49-64. (10.5565/rev/analisi.3398)

[114] Gao J, Hoffmann HF, Oikonomou S, Kiskovski D, Bandhakavi A. 2022 Logically at factify 2022: multimodal fact verification. In *Proceedings of CEUR Workshop on Multimodal Fact-Checking and Hate Speech Detection*, Vancouver, BC, Februrary 22 - March 1 2022, pp. 1-22. Aachen, Germany, Germany: CEUR-WS. (10.48550/arXiv.2112.09253)

[115] Bhattacharjee S.D., Yuan J. 2022. Multimodal Co-training for Fake News Identification Using Attention-aware Fusion. In Pattern Recognition. ACPR 2021. Lecture Notes in Computer Science, vol 13189. (eds C Wallraven, Q Liu, H Nagahara). Cham, Switzerland: Springer Nature. 10.1007/978-3-031-02444-3_21.

[116] Nielsen DS, McConville R. 2022 MuMiNA: large-scale multilingual multimodal fact-checked misinformation social network dataset. In *Proc. of the 45th Int. ACM SIGIR Conf. on Research and Development in Information Retrieval (SIGIR '22)*, Madrid, Spain, 11-15 July 2022, pp. 3141–3153. New York, NY: Association for Computing Machinery. 10.1145/3477495.3531744.

[117] Mehran W, Bayati UA, Mottet M, Lemieux AF. 2021 Deep analysis of Taliban videos: differential use of multimodal, visual and sonic forms across strategic themes. *Stud. Confl. Terror.* **0**, 1–21. (10.1080/1057610X.2020.1866739)

[118] Lee J, Shin SY. 2021 Something that they never said: multimodal disinformation and source vividness in understanding the power of AI-enabled deepfake news. Media Psychol. **25**, 531-546. (10.1080/15213269.2021.2007489)

[119] Choi H, Ko Y. 2022 Effective fake news video detection using domain knowledge and multimodal data fusion on youtube. Pattern Recognit. Lett. **154**, 44-52. (10.1016/j.patrec.2022.01.007)

[120] Shang L, Kou Z, Zhang Y, Wang D. 2021 A multimodal misinformation detector for COVID-19 short videos on TikTok. In *2021 IEEE Int. Conf. on Big Data* (*Big Data*), Orlando, FL, 15-18 December 2021, pp. 899–908. New York, NY: IEEE. (10.1109/BigData52589.2021.9671928)

[121] Zhou X, Mulay A, Ferrara E, Zafarani R. 2020 ReCOVery: a multimodal repository for COVID-19 news credibility research. In *Proc. of the 29th ACM Int. Conf. on Information & Knowledge Management* (CIKM '20)*,* Online, 19-23 October 2020, pp. 3205–3212. New York, NY: Association for Computing Machinery. (10.1145/3340531.3412880)

[122] Chen M, Chu X, Subbalakshmi KP. 2021 MMCoVaR: multimodal COVID-19 vaccine focused data repository for fake news detection and a baseline architecture for classification. In *Proc. of the 2021 IEEE/ACM Int. Conf. on Advances in Social Networks Analysis and Mining (ASONAM '21),* The Hague, Netherlands, 8-11 November 2022, pp. 31–38. New York, NY: Association for Computing Machinery. (10.1145/3487351.3488346)

[123] Elswah M, Howard PN. 2020 ‘Anything that Causes Chaos’: the organizational behavior of Russia today (RT). J. Commun. **70**, 623-645. (10.1093/joc/jqaa027)

[124] Li Y, Jiang B, Shu K, Liu H. 2020 Toward a multilingual and multimodal data repository for COVID-19 disinformation. In *2020 IEEE Int. Conf. on Big Data* (*Big Data*), Atlanta, GA, 10 December 2020, pp. 4325–4330. New York, NY: IEEE. (10.1109/BigData50022.2020.9378472)

[125] Sharma DK, Garg S. 2021 IFND: a benchmark dataset for fake news detection. *Complex Intell. Syst.* **9**, 2843-2863. 10.1007/s40747-021-00552-1 PMC8520332 34777983

[126] Luo G, Darrell T, Rohrbach A. 2021 NewsCLIPpings: automatic generation of out-of-context multimodal media. In *Proc. of the 2021 Conf. on Empirical Methods in Natural Language Processing*, Punta Cana, Dominican Republic, 8-11 November 2021, pp. 6801–6817. Stroudsburg, PA: Association for Computational Linguistics. (10.18653/v1/2021.emnlp-main.545)

[127] Singh VK, Ghosh I, Sonagara D. 2020 Detecting fake news stories via multimodal analysis. J. Assoc. Inform. Sci. Technol. **72**, 3-17. (10.1002/asi.24359)

[128] Wang Z, Yin Z, Argyris YA. 2021 Detecting medical misinformation on social media using multimodal deep learning. IEEE J. Biomed. Health Inform. **25**, 2193--2203. (10.1109/JBHI.2020.3037027) 33170786

[129] Wang Y, Ma F, Wang H, Jha K, Gao J. 2021 Multimodal emergent fake news detection via meta neural process networks. In *Proc. of the 27th ACM SIGKDD Conf. on Knowledge Discovery & Data Mining (KDD '21)*, Online, 14-18 August 2021, pp. 3708–3716. New York, NY: Association for Computing Machinery. (10.1145/3447548.3467153)

[130] Lv J, Wang X, Shao C. 2022 TMIF: transformer-based multi-modal interactive fusion for automatic rumour detection. *Multimedia Syst.* **29**, 2979-2989. (10.1007/s00530-022-00916-8)

